# An Interpretable Computer-Aided Diagnosis Method for Periodontitis From Panoramic Radiographs

**DOI:** 10.3389/fphys.2021.655556

**Published:** 2021-06-22

**Authors:** Haoyang Li, Juexiao Zhou, Yi Zhou, Qiang Chen, Yangyang She, Feng Gao, Ying Xu, Jieyu Chen, Xin Gao

**Affiliations:** ^1^Cancer Systems Biology Center, The China-Japan Union Hospital, Jilin University, Changchun, China; ^2^Computational Bioscience Research Center (CBRC), King Abdullah University of Science and Technology (KAUST), Thuwal, Saudi Arabia; ^3^College of Computer Science and Technology, Jilin University, Changchun, China; ^4^Department of Biology, Southern University of Science and Technology, Shenzhen, China; ^5^Department of Biochemistry and Molecular Biology and Institute of Bioinformatics, University of Georgia, Athens, GA, United States; ^6^The Affiliated Stomatological Hospital of Soochow University, Soochow, China; ^7^Department of Stomatology, The Sixth Affiliated Hospital, Sun Yat-sen University, Guangzhou, China; ^8^Department of Colorectal Surgery, The Sixth Affiliated Hospital, Sun Yat-sen University, Guangzhou, China

**Keywords:** teeth segmentation and numbering, periodontitis diagnosis, panoramic radiograph, computer-aided diagnostics, interpretable model

## Abstract

Periodontitis is a prevalent and irreversible chronic inflammatory disease both in developed and developing countries, and affects about 20–50% of the global population. The tool for automatically diagnosing periodontitis is highly demanded to screen at-risk people for periodontitis and its early detection could prevent the onset of tooth loss, especially in local communities and health care settings with limited dental professionals. In the medical field, doctors need to understand and trust the decisions made by computational models and developing interpretable models is crucial for disease diagnosis. Based on these considerations, we propose an interpretable method called Deetal-Perio to predict the severity degree of periodontitis in dental panoramic radiographs. In our method, alveolar bone loss (ABL), the clinical hallmark for periodontitis diagnosis, could be interpreted as the key feature. To calculate ABL, we also propose a method for teeth numbering and segmentation. First, Deetal-Perio segments and indexes the individual tooth via Mask R-CNN combined with a novel calibration method. Next, Deetal-Perio segments the contour of the alveolar bone and calculates a ratio for individual tooth to represent ABL. Finally, Deetal-Perio predicts the severity degree of periodontitis given the ratios of all the teeth. The Macro F1-score and accuracy of the periodontitis prediction task in our method reach 0.894 and 0.896, respectively, on *Suzhou* data set, and 0.820 and 0.824, respectively on *Zhongshan* data set. The entire architecture could not only outperform state-of-the-art methods and show robustness on two data sets in both periodontitis prediction, and teeth numbering and segmentation tasks, but also be interpretable for doctors to understand the reason why Deetal-Perio works so well.

## Introduction

**FIGURE 1:**
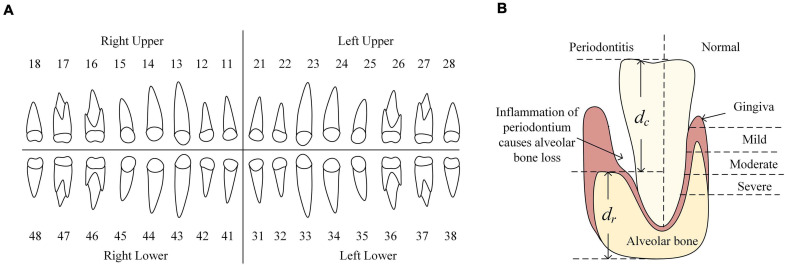
**(A)** FDI numbering system. It divides all teeth into four quadrants where teeth are labeled as 11–18, 21–28, 31–38, 41–48, respectively. **(B)** The left of tooth shows the appearance of periodontitis and the representation of ABL. d_*c*_ represents the ABL and d_*c*_ + d_*r*_ is used for normalize the ABL. Thus, each tooth has its ABL representation calculated as d_*c*_/(d_*c*_ + d_*r*_). The right of tooth shows the appearance of a normal tooth. Different severities of alveolar bone loss reflect the severities of periodontitis (normal, mild, moderate and severe periodontitis).

Periodontitis is a chronic inflammatory disease of periodontium resulting in inflammation within the supporting tissues of the teeth, progressive attachment, and bone loss (Lindhe et al., 1999). Periodontitis is prevalent in both developed and developing countries, and affects about 20–50% of the global population which makes it a public health concern (Nazir, 2017). Thus, the tool for automatically diagnosing periodontitis is highly demanded to provide the invaluable opportunity to screen at-risk people for periodontitis and its early detection could prevent the onset of tooth loss, especially in local community and health care settings where dentists are not easily accessible (Balaei et al., 2017). The form of periodontitis is characterized by periodontal ligament loss and resorption of the surrounding alveolar bone caused by severe inflammatory events (de Pablo et al., 2009). Cumulative alveolar bone loss (ABL) results in weakening of the supporting structures of the teeth, and predisposes the patient to tooth mobility and loss (Brunton, 2008; Figure 1B). Thus ABL is a hallmark of the periodontal disease (Yang et al., 2018). To calculate ABL of all teeth, it is necessary to gather the contours of the individual tooth and the alveolar bone. In this situation, teeth numbering and segmentation are essential and fundamental tasks for periodontitis diagnosis. In addition, dentists usually need to serve numerous patients and read a large number of panoramic radiographs daily. Thus an automatic tool for teeth numbering and segmentation to enhance efficiency and improve the quality of dental care is timely needed (Chen et al., 2019).

Several methods have been proposed to tackle the periodontitis prediction or teeth numbering and segmentation tasks. Joo et al. (2019) proposed a classification method for the periodontal disease based on convolutional neural network (CNN) by using periodontal tissue images. This method classified four states of periodontitis and the accuracy on validation data was 0.81. Ozden et al. (2015) tested three classification algorithms, artificial neural networks (ANN), support vector machine (SVM), and decision tree (DT) to diagnose periodontal diseases by using 11 measured variables of each patient as raw data. The results showed that DT and SVM were best to classify the periodontal diseases with high accuracy (0.98 of precision). It revealed that SVM and DT appeared to be practical as a decision-making aid for the prediction of periodontal disease. Lee et al. (2018) proposed a periodontitis prediction method using periapical radiographic images via deep CNN. The diagnostic accuracy for periodontally compromised teeth was 81.0% for premolars and 76.7% for molars. Li et al. (unpublished) proposed a method which could screen the existence of gingivitis and its irritants, i.e., dental calculus and soft deposits, from oral photos with a novel multi-task learning model. With 625 patients included in this study, the classification area under the curve for detecting gingivitis, dental calculus and soft deposits were 87.11, 80.11, and 78.57%, respectively.

As for the teeth numbering and segmentation, Wirtz et al. (2018) proposed a coupled shape model in conjunction with a neural network by using panoramic radiographs. The network provided a preliminary segmentation of the teeth region which is used to initialize the coupled shape model. Then the 28 individual teeth (excluding wisdom teeth) were segmented and labeled using gradient image features in combination with the model’s statistical knowledge. The experimental result showed an average dice of 0.744. Chen et al. (2019) used faster regions with CNN features to detect and number teeth in dental periapical films. They proposed three post-processing techniques to improve the numbering performance. Results revealed that mean average precision (mAP) was 0.80 and the performance of this method was close to the level of junior dentists. Cui et al. (2019) used deep CNN to achieve automatic and accurate tooth instance segmentation and identification from cone-beam CT (CBCT) images. They extracted the edge map from the CBCT image to enhance image contrast along shape boundaries. Next, the edge map and input images were passed through 3D Mask R-CNN with encoded teeth spatial relationships. Their method produced accurate instance segmentation and identification results automatically.

The main limitations of methods mentioned above are as follows: (1) the bias of detecting and numbering teeth in some cases with severe periodontitis due to the disturbance of a large number of missing teeth, (2) the lacking capability of their methods on predicting the number of missing teeth in the shortage data volume of some individual classes, and (3) the lack of interpretability of predicting the severity degree of periodontitis.

In this paper, we try to overcome these limitations through the following contributions. (1) We propose an automatic and interpretable method called Deetal-Perio to predict the severity degree of periodontitis from dental panoramic radiographs. (2) As a subroutine of Deetal-Perio, we further propose a method for teeth numbering and segmentation which consists of a novel calibration algorithm. (3) Deetal-Perio outperforms state-of-the-art methods and shows the robustness on the two data sets from two hospitals. (4) Deetal-Perio uses ABL as the feature for periodontitis diagnosis and is thus fully interpretable.

We note that a shorter conference version of this paper appeared (Li et al., 2020b). This manuscript added more comprehensive details of data annotation, the calibration method for teeth numbering, implementations of experiments and measurements. We also collected more in-house data from the cooperative hospital to enhance the performance of our method.

## Materials and Methods

### Data Sets

The Affiliated Stomatological Hospital of Soochow University supplied a total of 302 digitized panoramic radiographs (hereinafter referred to as the *Suzhou* data set). Each radiograph has a high resolution of 1,480 ^∗^ 2,776 pixels and was annotated following the Fédération Dentaire Internationale (FDI) numbering system to get the contours of teeth and their labels as the ground truth (GT). FDI numbering system divides all teeth into four quadrants where teeth are labeled as 11–18, 21–28, 31–38, 41–48, respectively (Figure 1A). Among all radiographs, 298 were labeled with the severity degree of periodontitis by dentists, including four categories: 52 of no periodontitis, 189 of mild periodontitis, 43 of moderate periodontitis, and 14 of severe periodontitis. The 4 missing images contain the mislabeled images and unlabeled images.

We also collected another data set from the Sixth Affiliated Hospital, Sun Yat-sen University (hereinafter referred to as the *Zhongshan* data set). This data set includes 204 panoramic radiographs with high and various resolution which are categorized by four classes mentioned above (69 of no periodontitis, 54 of mild periodontitis, 42 of moderate periodontitis, and 39 of severe periodontitis).

These two data sets are both in-house data sets which are created by the machines called SOREDEX DIGORA Optime from KaVo Dental (North Carolina, United States) and GiANO from NewTom (Bologna, Itlay), respectively. Each data set was labeled by one expert dentist with more than 10 years of clinical experience, respectively. The pixel value in the radiograph reflected by the density (the quantity of darkness). The procedure of annotation is as follows. Given a panoramic radiograph, the dentist would draw a contour along with the boundary of each existing tooth and assign a corresponding tooth number to each existing tooth by following the FDI numbering system. Next, the dentist assigned the stage of periodontitis for each radiograph according to the visualizing of radiograph and the recognized standard from previous study (Papapanou et al., 2018). Here we also define a ratio c for each tooth, which is defined as the distance between cemento-enamel junction (CEJ) and alveolar bone divided by the distance between CEJ and the dental root, to indicate the level of bone loss for each tooth. No periodontitis is defined that none of teeth has bone loss. Mild periodontitis is defined that at least the c of one tooth is less than 15%. Moderate periodontitis is defined that at least the c of one tooth is less than 33% and larger than 15%. Severe periodontitis is defined that at least the c of one tooth is larger than 33%.

### Methods

**FIGURE 2:**
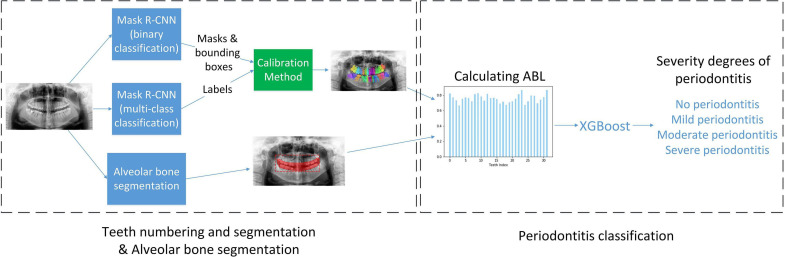
The workflow of Deetal-Perio.

The architecture of Deetal-Perio is as follows. First, Deetal-Perio segments and numbers individual tooth via Mask R-CNN combined with a novel calibration method. Next, Deetal-Perio segments the contour of the alveolar bone and calculates the ratio for individual tooth which could represent ABL as the key feature to predict periodontitis. Finally, Deetal-Perio uses XGBoost to predict the severity degree of periodontitis by given a vector of ratios from all the numbered teeth. The entire architecture is shown in Figure 2.

#### Teeth Segmentation and Numbering

**FIGURE 3:**
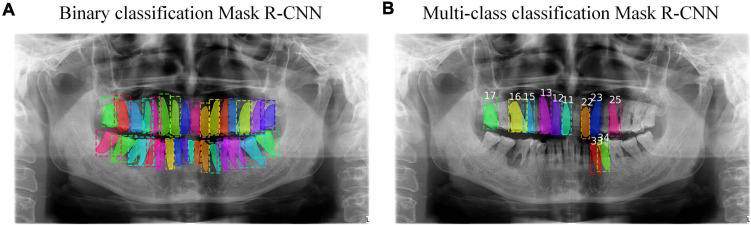
**(A)** The result of binary classification Mask R-CNN. **(B)** The result of multi-class classification Mask R-CNN. Each color in these two results represents different instances.

Inspired by the state-of-the-art architecture in object classification and segmentation called Mask R-CNN (Abdulla, 2017; He et al., 2017), we tried to segment the teeth with binary classification via Mask R-CNN. That was, we wanted to differentiate teeth from the background image. The result revealed that Mask R-CNN could detect almost all of the teeth given a radiograph. Then, we tried to segment and number the teeth with multi-class classification via Mask R-CNN and this time, we wanted to identify the labels of these teeth. The result showed that only a minority of teeth could be detected and numbered due to the limitation of data from individual classes, but compared with the GT, most detected teeth were numbered correctly. Figure 3 shows these two results, which provide complementary information to each other. Thus, we proposed to combine the results from the binary and multi-class Mask R-CNN models together. We extracted the bounding boxes and masks from the binary classification results, and the labels of numbered teeth from the multi-class classification results. We further proposed a calibration method to integrate the results from the two classifiers, refine the labels of numbered teeth, and infer the labels of unnumbered teeth.

**FIGURE 4:**
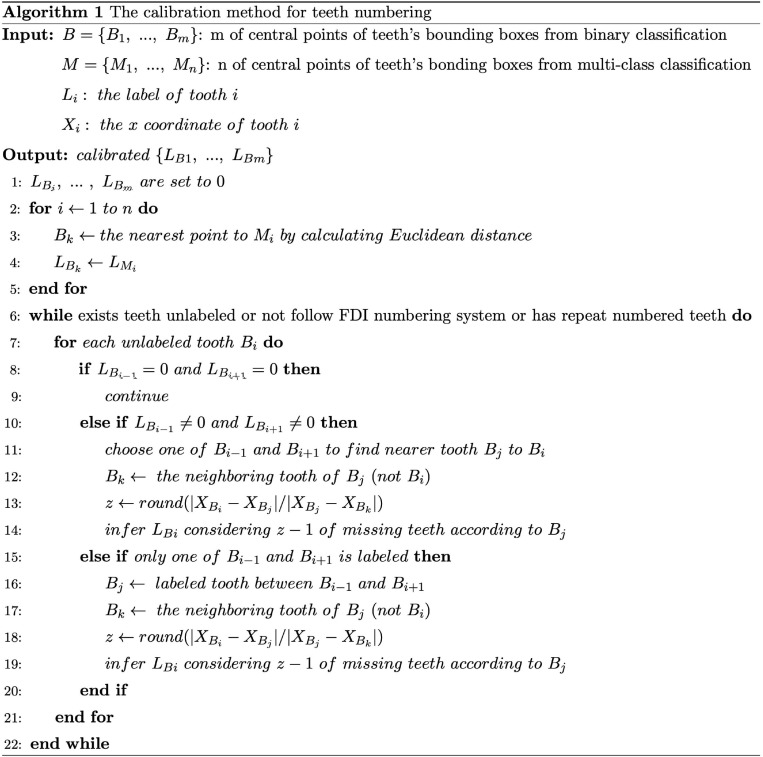
The calibration algorithm for teeth numbering.

The calibration method is designed as follows: first, B={*B*_1_, *B*_2_, …,*Bm*} and M={*M*_1_,*M*_2_, …,*Mm*} represent m and n of center points of teeth’ bounding boxes from the results of binary and multi-class classification Mask R-CNN, respectively. Then, we found the closest tooth to each tooth of M in B by calculating the Euclidean distance and assigned the labels of teeth from M to B. Next, we iterated each tooth in B to judge whether neighboring teeth are labeled and calibrated its own label until all the teeth in B satisfied three conditions: 1. each tooth had been labeled; 2. no repeatedly labeled tooth; 3. all labeled teeth followed the rules of FDI numbering system. During the iteration of unlabeled teeth, there would be three circumstances. First, if neither of the neighboring teeth was labeled, we would skip. Second, if one of the neighboring teeth was labeled, we would use the ratio of distances between adjacent teeth to inference the label of selected tooth. For example, we selected tooth B_*i*_ after several iterations and the right neighboring of B_*i*_, called B_*i*+1_, had been numbered. We defined D_*r*_ as the horizontal distance from B_*i*_ to B_*i*+1_, D_*rr*_ as the horizontal distance from B_*i*+1_ to the right neighboring tooth of B_*i*+1_. Next, we calculated the rounded ratio *D_r_*/*D_rr_* which indicates the number of teeth between B_*i*_ and B_*i*+1_. Then, this rounded ratio could be utilized to inference the label of B_*i*_ according to B_*i*+1_. Third, when both of neighboring teeth were labeled, it is similar to the previous circumstance that we used the nearer neighbor of selected tooth and its adjacent tooth to inference the label of selected tooth. For example, we also defined the selected tooth as B_*i*_, the nearer neighboring tooth of B_*i*_ as B_*j*_, and the neighboring tooth of B_*j*_ as B_*k*_ (not B_*i*_). Similarly, we utilized the rounded ratio, the distance from B_*i*_ to B_*j*_ divided by the distance from B_*j*_ to B_*k*_, to indicate the number of teeth between B_*i*_ and B_*j*_, which could be used to inference the label of B_*i*_. The details of algorithm are given in Figure 4. Finally, all teeth are labeled in B which are considered as the results of teeth segmentation and numbering step.

#### The Representation of ABL

As previously introduced, ABL results in weakening of the supporting structures of the teeth, which makes it the hallmark of periodontal disease (Brunton, 2008; Figure 1B) and the annotation of degrees of periodontitis also depends on the severities of ABL from all of teeth. Thus, we were going to use the contours of individual teeth and their labels we have obtained and the contour of the alveolar bone to calculate the severity of ABL for each individual tooth. We used binary classification Mask R-CNN to segment the contour of alveolar bone and the confidence scores of detected boxes are high in the results. Crown-to-Root Ratio (CRR) is a kind of prognostic parameter in periodontology, which could be defined as, CRR=*d_c_*/*d_r_* where *d_c_* denotes the vertical distance from the alveolar bone to the top of the dental crown and denotes the vertical distance from the bottom of the dental root to the alveolar bone (Figure 1B). We also defined the severity of ABL as the ratio d=*CRR*/(*1+CRR*) for each individual tooth. Due to the smoothness of the contour of the alveolar bone, we randomly selected two points on this contour to draw a line. Then, we randomly chose 50% points on the contour of the dental crown to calculate the vertical distance from these points to this line, respectively, and defined the largest vertical distance as d_*c*_. We estimated d_*r*_ in a similar way. In our case, d_*c*_ could be a good estimation to represent ABL showing the level of destruction of the alveolar bone and to normalize d_*c*_, we divided it by *d_c_* + *d_r_* which is the estimation of the perpendicular height of the tooth. Thus, d could represent the ABL of each tooth.

#### Periodontitis Prediction

After acquiring the ratio d of individual tooth, each radiograph would output a vector of ratios D={*d*_1_,*d*_2_…,*d*_32_} where each radio corresponds to a label of tooth. Apparently, some radiographs do not have all the 32 teeth. In such cases, the ratios of teeth which do not exist in the radiograph are set to be the mean value of its neighboring teeth’ ratios. We then solved the periodontitis classification task by XGBoost (Chen and Guestrin, 2016) which has gained attention as the algorithm of choice for many winning teams of machine learning competitions (Volkovs et al., 2017). To tackle the class imbalance problem, we used Synthetic Minority Over-sampling Technique (SMOTE) (Chawla et al., 2002) for over-sampling the minority classes. After over-sampling, *D* was inputted as the feature to classify the four-class severity degree of periodontitis by XGBoost.

## Results

### Experimental Setup

The binary and multi-class classification of Mask R-CNN were trained starting from pre-trained COCO weight and fine-tuned on our panoramic radiographs. We used ResNet-101 as the backbone network to extract features and Mask R-CNN was implemented for 30 epochs with a learning rate of 0.001 to avoid making the learning jump over minima and the batch size was set to 1. We also configured the minimum confidence score of detection as 0.7, because there were several teeth with abnormal shapes and we did not want to eliminate them. The threshold of non-maximum suppression was set to 0.3 to guarantee enough proposals retained and allow the existence of an overlap between teeth. During the data pre-processing, we resized all the panoramic radiographs to 1,024 ^∗^ 1,024 pixels. We preserved the previous ratio, which means if an image was not square, we would pad it with zeros. All experiments were conducted by 2 NVIDIA QUADRO M6000 24GB GPU. The methods were running backend on TensorFlow version 1.9.0 and operating system was Ubuntu 16.04.

We randomly extracted 80% (i.e., 241) and 80% (i.e., 163) of *Suzhou* and *Zhongshan* data sets, respectively, as two training sets and the rest 20% (i.e., 61) and 20% (i.e., 41) were two testing sets, respectively. The reason why we didn’t choose the validation test is to take full advantage of our collected data. We trained Mask R-CNN starting from pre-trained COCO weight on *Suzhou* training set and then we fine-tuned this model on *Zhongshan* training set. Finally, we tested the performance of our model on two testing sets, respectively. All training and testing processes have been repeated three times, and the final results were averaged from these three experiments. For comparison with other methods, we applied the same training and testing procedure on our dataset as we did with our method.

### Performance

**FIGURE 5:**
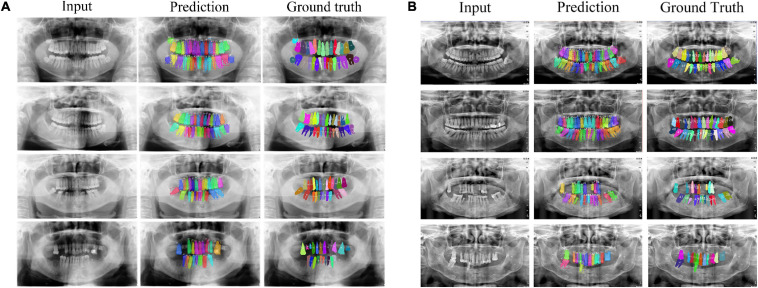
Teeth numbering and segmentation results tested on *Suzhou* and *Zhongshan* data set are shown in subplot **(A,B)**, respectively. From top to bottom cases for each panel, they are no periodontitis, mild periodontitis, moderate periodontitis and severe periodontitis.

Figures 5A,B shows eight examples of teeth segmentation and numbering, the two panels include the results of four kinds of periodontitis from the *Suzhou* data set and *Zhongshan* data set, respectively, showing good performance and robustness in both datasets. Table 1 shows the comparison of our teeth segmentation and numbering method with the baseline Mask R-CNN method and those proposed by Wirtz et al. (2018) and Chen et al. (2019) on our two datasets. The comparison is quantified by three metrics: (1) Dice (all) denotes the overall dice coefficient of teeth segmentation, which represents the capability of our method to detect and segment the teeth. We evaluated Dice (all) after the implementation of binary classification Mask R-CNN, thus Dice (all) could be defined as:

**TABLE 1:** Performance comparison tested on the *Suzhou* data set and *Zhongshan* data set between Deetal-Perio and other methods on teeth numbering/segmentation task, by mAP, Dice (all) and Dice (single).

	***Suzhou* data set**			***Zhongshan* data set**		
**Methods**	**mAP**	**Dice (all)**	**Dice (single)**	**mAP**	**Dice (all)**	**Dice (single)**
**Deetal-Perio**	**0.863**	**0.892**	**0.809**	**0.927**	**0.903**	**0.819**
Multi-class Mask R-CNN	0.834	0.830	0.781	0.881	0.869	0.801
Wirtz et al. (2018)	0.435	0.765	0.502	0.409	0.648	0.428
Chen et al. (2019)	0.680	–	–	0.559	–	–

*The method of Chen et al. (2019) only has tooth numbering, but not segmentation function. The best performance in each category is in bold.*

(1) $$Dice\left(all\right)=\frac{2\left|X\cap Y\right|}{\left|X\right|+\left|Y\right|}$$

X represents the binary mask of prediction for all the teeth and the Y denotes the binary mask of GT for all the teeth. (2) The mAP denotes the capability of whether our method detects all numbers of teeth correctly, which is calculated as follows. First, we choose the proposal, whose intersection over union (IoU) with GT of a specific class is higher than the pre-defined threshold (normally set to 0.5) and the confidence score is the highest, as the true positive and the other proposals corresponding the same GT would be defined as false positive. Thus, we could calculate the precision, recall, and precision-recall curve for a particular class. The area under the precision-recall curve is the average precision (AP) of this specific class and the mAP could be calculated as the mean value of the APs for all object classes. (3) Dice (single) denotes the mean value of all dice coefficients from all labeled teeth, respectively, which could evaluate the performance of our method to segment each specific tooth. Dice (single) could be defined as:

(2) $$Dice\left(single\right)=\sum_{i=0}^{n}Dice\left(P_{i},G_{i}\right)/n$$

where n represents the number of teeth in the GT, P_*i*_ and G_*i*_ represent the i-th mask of a specific tooth from prediction and GT, respectively. Thanks to the calibration method in Deetal-Perio, we could number teeth much more correctly than other compared methods. Thus, the performance of Deetal-Perio in the segmentation of individual tooth is also better than other methods. In the compared methods, Chen et al. (2019) focus on the teeth numbering task, which output the mAP only. Their method is not trained on panoramic radiographs like ours, but the dental periapical films. Thus, their post-processing method may not work well on our data sets, which causes a worse performance than ours. The method of Wirtz et al. (2018) requires the fixed image size to inference the contour for each tooth which could not work on our data sets, which includes various sizes of panoramic radiographs. Less of robustness of their method for image size causes a worse performance than ours. Comparing the two datasets, we could also find that the results from *Zhongshan* data set are a little better than the results from *Suzhou* data set, and this could be caused by the different quality of the radiographs from the two hospitals. The radiographs from *Zhongshan* data set are more enhanced than *Suzhou*’s, including the darker background and the brighter foreground, which could make it easier for the network to extract dental features.

We then evaluated the performance of periodontitis prediction. Table 2 compares our method with several baseline machine learning methods and the method proposed by Joo et al. (2019) on our two data sets. The input of Joo et al. (2019) is the resized panoramic radiograph and the other compared methods accept the ABL vector as the input. The metrics include Macro F1 score and accuracy over the four classes, which are defined as following equations:

**TABLE 2:** Performance comparison tested on the *Suzhou* data set and *Zhongshan* data set between Deetal-Perio and five methods by F1-score and accuracy on the periodontitis prediction task.

	***Suzhou* data set**		***Zhongshan* data set**	
**Methods**	**Macro F1-score**	**Accuracy**	**Macro F1-score**	**Accuracy**
**Deetal-Perio**	**0.889**	**0.892**	**0.812**	**0.819**
SVM	0.693	0.711	0.449	0.590
Decision tree	0.745	0.758	0.643	0.665
Adaboost	0.701	0.742	0.670	0.688
CNN	0.591	0.611	0.669	0.729
Joo et al. (2019)	0.331	0.408	0.318	0.367

*The best performance in each category is in bold.*

(3) $$MacroF1score=\sum_{i=0}^{3}\frac{2\times \left(P_{i}\times R_{i}\right)}{P_{i}+R_{i}}/4$$

(4) $$Accuracy=\frac{n_{correct}}{n_{total}}$$

where **P**_*i*_ and **R**_*i*_ denote the precision and recall of the i-th class, n_*correct*_ indicates the number of prediction classified correctly, and n_*total*_ denotes the number of total data. Because the class-imbalanced issue has been solved by SMOTE, the usage of accuracy makes sense to evaluate the performance of our method. Considering the balance between precision and recall of each class, we use Macro F1 score to evaluate our method. The reason why we choose XGBoost as our classifier is that the boosting algorithm of XGBoost made it a strong learner to enhance the performance compared with the simple decision trees, and the regularization of XGBoost made it robust against the noise and thus outperforming Adaboost. The reason why CNN does not have great performance is that CNN may bring the overfitting issue when handling such low dimension input data. Compared to Joo et al. (2019), our method greatly reduces the feature dimension from a radiograph to a 1 × 32 vector instead of simply implementing a CNN for image classification with a large number of disturbing redundant features. In addition, the intermediate results of teeth segmentation and numbering as well as the geometrically calculated ABL provide dentists completely refined and interpretable information, so that they not only know that our method works, but also understand how it works.

## Discussion and Conclusion

In this paper, we proposed a fully automatic and completely interpretable method, Deetal-Perio, for diagnosing the severity degrees of periodontitis from panoramic radiographs using ABL as the key feature. As the intermediate results, our method also accomplished teeth numbering and segmentation tasks. Comprehensive experiments on two data sets show that Deetal-Perio not only dramatically outperforms other compared methods in both teeth segmentation and numbering, and periodontitis prediction, but is also robust and generalizable on independent data sets, which makes Deetal-Perio a suitable method for periodontitis screening and diagnostics.

**FIGURE 6:**
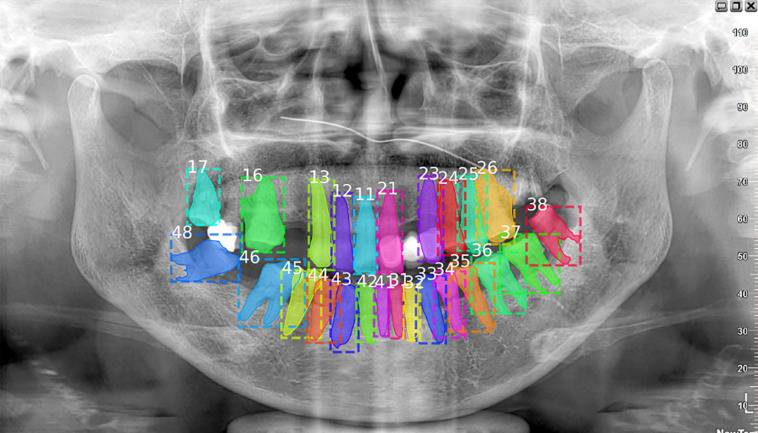
This case missed the filled tooth between number 17 and 16. Actually, the missing tooth should be number 17 and the tooth which is wrongly numbered as 17 should be 18. Considering about the filled teeth could enhance the performance of teeth numbering and segmentation tasks.

Despite the success of Deetal-Perio, the performance of teeth numbering relies on the numbering results from the multi-class classification Mask R-CNN model in Deetal-Perio. This can cause issues when there are radiographs with severe periodontitis which have only few or abnormal shapes of teeth. To overcome this limitation, more data, similar to these special cases, is needed to further improve the performance, employing few-shot learning might be a helpful way to deal with such special situations (Li et al., 2019, 2020a). We also found that there are filler materials in some teeth which could not be detected (Figure 6). Although these are a minority of cases, we need to focus on them in order to enhance the performance of teeth segmentation and numbering results in our model. We also plan to develop some functions, such as detecting the filled teeth automatically based on our model, which could assist the dentists during diagnosis at a higher level.

From the clinical perspective, the comprehensive process of periodontitis diagnosis should be also based on the measures like clinical attachment loss (CAL) which is costly for us to collect. For better performance and applicability of our method, we plan to collect this kind of data and integrate them with our method to make it more convincing for clinicians.

## Data Availability Statement

The raw data supporting the conclusions of this article will be made available by the authors, without undue reservation.

## Author Contributions

HL implemented the pipeline, did the experiments, evaluated the performance, and wrote the manuscript. JZ designed the calibration method and did that part of experiments. YZ preprocessed raw data. QC, YS, JC, and FG supplied the in-house data sets. XG and YX supervised the study and wrote the manuscript. All authors contributed to the article and approved the submitted version.

## Conflict of Interest

The authors declare that the research was conducted in the absence of any commercial or financial relationships that could be construed as a potential conflict of interest.
